# Silent neurovestibular frailty: a conceptual model integrating vestibular dysfunction, dual-task mobility and cognitive load as candidate contributors to falls, delirium and functional decline in older adults

**DOI:** 10.3389/fnagi.2026.1879751

**Published:** 2026-08-19

**Authors:** Samaher Mohammed Alowaydhah, Ugwu Okechukwu Paul-Chima, Abdulmajeed Alfayyadh, Sulaiman Alanazi

**Affiliations:** 1 Department of Physiotherapy and Health Rehabilitation, College of Applied Medical Sciences, Jouf University, Sakaka, Saudi Arabia; 2 Department of Research and Publication, Kampala International University, Kampala, Uganda; 3 Department of Physical Therapy and Health Rehabilitation, College of Applied Medical Sciences, Jouf University, Sakaka, Saudi Arabia

**Keywords:** ageing, conceptual model, delirium, dual-task gait, falls, frailty, functional decline, neurovestibular

## Abstract

Falls, delirium and functional decline are commonly treated as distinct geriatric outcomes despite sharing vulnerabilities in sensory integration, gait regulation, attention and physiological reserve. We propose silent neurovestibular frailty as a conceptual model describing a latent ageing phenotype in which vestibular dysfunction, dual-task gait deterioration and sensitivity to cognitive load interact before overt disability becomes apparent. This construct is presented as a hypothesis-generating framework rather than a validated diagnostic entity, intended to integrate currently separate lines of evidence into a testable model. Vestibular impairment in later life is associated with balance deficits, mobility limitation and reduced functional reserve, whilst dual-task gait assessment may reveal cognitive-motor interference that remains undetected during routine walking evaluation, although dual-task cost may reflect impairment, strategic task prioritisation, or both. Delirium introduces additional clinical urgency because acute cognitive deterioration frequently develops in older adults with sensory impairment, immobility, multimorbidity, medication exposure and diminished adaptive reserve. We argue that vestibular function, dual-task mobility and cognition deserve coordinated assessment as a candidate translational risk triad for older adults vulnerable to falls, hospital-acquired delirium and accelerated functional decline, whilst recognising that prospective evidence validating this integrated phenotype is currently lacking. The model extends rather than replaces multifactorial fall prevention and provides an empirical agenda for operationalisation, validation and intervention testing across community, perioperative, inpatient and rehabilitation settings.

## Introduction

Population ageing has increased the need to identify older adults before falls, delirium or loss of independence occur. These outcomes are commonly managed in separate clinical pathways, although they frequently coexist and reinforce one another. A fall may precipitate fear, inactivity, fracture, admission, deconditioning and institutionalisation, whilst delirium can complicate acute illness or surgery and is associated with prolonged admission, cognitive decline, functional deterioration and mortality (Inouye et al., 2014; Oh et al., 2017). This overlap suggests that geriatric assessment should move beyond parallel evaluation of balance, cognition, sensory function and mobility, and should instead examine how these systems interact under stress.

The World Guidelines for Falls Prevention and Management for Older Adults recommend multifactorial risk stratification, gait and balance assessment, medication review, visual assessment, cardiovascular evaluation, cognitive assessment and targeted exercise interventions (Montero-Odasso et al., 2022). Exercise and multifactorial interventions remain central to prevention in community-dwelling older adults (Gillespie et al., 2012; Sherrington et al., 2019). However, assessment under simple clinical conditions may miss older adults who walk adequately in a corridor but destabilise when sensory input is degraded, attention is divided or the environment becomes crowded, dark, uneven or unfamiliar.

We introduce silent neurovestibular frailty as a candidate conceptual model for this hidden vulnerability, developed to organise otherwise separate observations from vestibular ageing, dual-task gait and delirium research into a single, testable framework rather than to assert a new diagnostic category. The term describes a latent phenotype in which vestibular dysfunction, cognitive-motor interference and reduced cognitive reserve may jointly compromise mobility and acute brain resilience. The phenotype is proposed as silent because it may not be detected by usual gait speed, a single bedside balance task or brief cognitive screening; the evidence for this detection gap, however, is presently inferential rather than directly demonstrated for the combined triad. It is termed neurovestibular because vestibular signals contribute to gaze stabilisation, balance, spatial orientation, navigation and multisensory integration. It is described using the language of frailty because it is hypothesised to reflect diminished reserve across interacting systems and increased vulnerability to disproportionate adverse outcomes after modest stressors; this use of ‘frailty’ is conceptual rather than a claim of formal frailty-syndrome status, a distinction developed further below.

The concept draws on evidence that vestibular dysfunction is common and functionally important in older adults (Agrawal et al., 2009; Lin and Bhattacharyya, 2012), that gait dysfunction can precede cognitive decline and dementia (Verghese et al., 2007), and that cognitive impairment increases fall risk (Muir et al., 2012). It is also consistent with reviews showing that cognition contributes to balance, gait and dual-task performance in ageing (Li et al., 2018), and with studies demonstrating impaired dual-task performance in older adults with mild cognitive impairment and mild Alzheimer’s disease (Ansai et al., 2017; Goncalves et al., 2018). None of this literature, however, has tested vestibular status, dual-task gait and delirium vulnerability together as a single construct, and the present Perspective should be read as an argument for why that integration is worth testing, not as a report that it has already been validated.

## Conceptual status: what kind of construct is silent neurovestibular frailty?

Before describing its components, it is necessary to clarify what silent neurovestibular frailty is intended to be, because imprecision on this point risks the construct being mistaken for an established diagnostic entity. We do not propose it as a subtype of physical frailty in the sense used by standard frailty phenotypes, nor as a diagnostic entity with defined thresholds. We propose it instead as a risk phenotype and organising heuristic that groups three measurable, already-studied vulnerabilities vestibular, dual-task mobility and cognitive-load under a single testable hypothesis, namely that their combination identifies older adults at elevated risk of falls, delirium and functional decline beyond what any one domain predicts alone. This is closer in status to motoric cognitive risk syndrome or cognitive frailty at the point those constructs were first proposed than to a validated clinical tool, and like those constructs it will require formal operational criteria, reliability testing and prospective validation before it can be used for risk stratification in practise.

We further distinguish two possible uses of the model that should not be conflated. As a mechanistic framework, it proposes candidate physiological pathways sensory reweighting, attentional cost of balance, and reduced adaptive reserve linking vestibular ageing to gait and cognitive vulnerability. As a screening heuristic, it proposes that clinicians consider vestibular symptoms, dual-task gait and cognitive vulnerability together during fall-risk assessment even before formal validation is complete, because each component already has an independent evidence base and the practical cost of joint enquiry is low. The mechanistic and heuristic uses require different kinds of evidence: the former needs physiological and neuroimaging studies, the latter needs prospective predictive-validity studies against outcomes such as falls, delirium and functional decline. We keep this distinction explicit throughout rather than presenting the model as an established phenotype.

## Relationship to related constructs

Silent neurovestibular frailty overlaps with, but is not equivalent to, several established constructs, and clarifying these boundaries is necessary to situate the proposed model accurately within existing frameworks.

Cognitive frailty describes the co-occurrence of physical frailty and cognitive impairment without dementia (Kelaiditi et al., 2013). It does not incorporate a sensory or vestibular component, and its physical-frailty criterion (e.g., slowness, weakness, exhaustion) is broader than the specific attentional and gait-interference measures emphasised here.

Motoric cognitive risk syndrome links slow gait with cognitive complaints as a dementia-risk marker (Xiang et al., 2022). It is defined primarily from usual-pace gait speed and subjective cognitive complaint, and does not require dual-task testing or vestibular assessment; silent neurovestibular frailty instead foregrounds gait performance under attentional load rather than usual-pace gait alone, and adds vestibular status as a third, independently measurable domain.

Sensory frailty concepts in the literature have generally emphasised vision and hearing loss as contributors to disability and cognitive decline, with vestibular function addressed less consistently. Silent neurovestibular frailty places the vestibular system at the centre of the sensory contribution rather than treating it as one item within a broader sensory-impairment count.

The dual-task gait literature has established that cognitive-motor interference predicts falls in populations with cognitive impairment (Ansai et al., 2017; Goncalves et al., 2018), but this literature has not generally incorporated vestibular status as a co-factor, nor extended prediction to delirium. Falls in older adults arise from interacting neurological, sensory, cognitive, musculoskeletal and environmental risk factors (Lord et al., 2007; Fasano et al., 2012).

Multifactorial fall-risk models (Montero-Odasso et al., 2022) already include gait, balance, vision, cardiovascular and medication assessment, and remain the appropriate foundation for practise; the contribution proposed here is a specific subgroup lens: one that treats vestibular dysfunction and dual-task gait interference as a coupled mechanism worth assessing together, and that extends this coupling towards delirium risk, which multifactorial models do not typically address.

The novel element of silent neurovestibular frailty is therefore not any single domain but the explicit joint assessment of vestibular status, attention-demanding dual-task gait, and cognitive-load vulnerability as one interacting unit linked to both falls and delirium. This joint linkage, to our knowledge, has not been tested directly and is the principal claim requiring empirical study, not an established finding.

## Defining silent neurovestibular frailty

Frailty is generally understood as reduced physiological reserve and increased vulnerability to stressors. Motoric cognitive risk syndrome and cognitive frailty have extended this idea by linking slow gait, cognitive complaints, dementia risk and recovery trajectories, but neither construct treats vestibular function as a core contributor (Kelaiditi et al., 2013; Xiang et al., 2022), a gap that silent neurovestibular frailty is intended to address conceptually rather than to have already closed empirically. This omission matters because the vestibular system is a principal sensory system for orientation in space, gaze stability, balance control and postural adaptation. Age-related vestibular loss may remain undiagnosed when older adults report nonspecific unsteadiness, reduced confidence, visual motion sensitivity or activity restriction rather than classic rotational vertigo (Agrawal et al., 2009; Lin and Bhattacharyya, 2012).

We propose that silent neurovestibular frailty can be understood, provisionally, as the interaction of three measurable and independently supported vulnerabilities. Vestibular vulnerability includes reduced vestibulo-ocular reflex function, impaired canal or otolith signalling, dizziness, oscillopsia, imbalance, visual dependence and spatial disorientation. Dual-task mobility vulnerability is reflected by slowing, increased gait variability, reduced stride regularity, turning instability, truncal sway or poorer obstacle negotiation when an attention-demanding task is added, interpreted alongside the task-prioritisation caveats discussed below. Cognitive-load vulnerability is reflected by impairment in attention, executive function, processing speed, visuospatial ability or fluctuation during acute stress. We emphasise that the co-occurrence of these three vulnerabilities as a single predictive unit is the hypothesis under discussion, not a demonstrated empirical pattern.

An older adult with mild vestibular hypofunction may compensate whilst walking along a straight, well-lit corridor, but may become unstable when navigating a busy clinic, turning quickly, walking in darkness, talking whilst walking or recovering from anaesthesia. Similarly, a patient with mild executive dysfunction may perform adequately during a brief cognitive screen but develop delirium during infection, dehydration, sleep disruption or psychoactive medication exposure (Inouye et al., 2014; Oh et al., 2017). The construct is intended to capture this plausible mismatch between controlled clinical performance and real-world vulnerability, a mismatch that is clinically familiar but has not yet been quantified for the specific triad proposed here.

## The brain gait vestibular axis

Walking in later life is not a purely automatic motor act. It depends on executive function, attention, sensory reweighting, anticipatory control and rapid correction of instability. Because these terms are used loosely across the literature, we define them as used here. Cognitive load refers to the momentary demand placed on attentional and executive resources by a concurrent task or environment. Cognitive reserve refers to the capacity of an individual’s neural and cognitive systems to sustain performance despite pathology or increased demand, and is a trait-like property rather than a momentary state. Attentional allocation refers to how finite attentional resources are distributed between competing tasks, such as walking and a concurrent cognitive task. Compensatory control refers to the deliberate or automatic recruitment of additional cognitive or postural resources to maintain performance when normal control mechanisms are degraded. These distinctions matter because a person can have adequate cognitive reserve yet still show high momentary cognitive load and gait interference under specific dual-task conditions, and the reverse mismatch is equally possible.

The vestibular system contributes by stabilising gaze, informing head and body orientation, supporting postural reflexes and interacting continuously with visual and somatosensory inputs. With ageing, degraded vestibular input may increase dependence on vision, proprioception and conscious attentional control, a compensatory shift that, we hypothesise, raises the cognitive load of otherwise automatic gait. When these compensatory systems are also impaired, gait stability becomes fragile, particularly during multitasking or environmental complexity (Lacour et al., 2008; Li et al., 2018).

The cognitive consequences of vestibular dysfunction are increasingly recognised. Reviews have linked vestibular disorders with impairments in attention, visuospatial processing, spatial memory, executive function and navigation (Bosmans et al., 2021; Smith et al., 2024). Vestibular loss in older adults has also been associated with impaired spatial navigation on the triangle completion task (Xie et al., 2017). These cognitive domains are plausibly relevant to safe mobility because older adults must judge distance, direction, speed, lighting, surface conditions and moving obstacles whilst walking, although the studies cited here examined vestibular-cognitive associations directly and did not test the combined predictive value proposed in this Perspective.

The same axis offers a plausible, though still unproven, bridge between falls and delirium. Delirium occurs when acute stressors overwhelm vulnerable neural systems, and recognised risk factors include cognitive impairment, sensory impairment, immobility, pain, infection, dehydration, sleep disruption and medication exposure (Inouye et al., 2014; Oh et al., 2017). Vestibular dysfunction may contribute through disorientation, visual dependence, motion sensitivity, postural insecurity and activity restriction. Direct prospective evidence linking vestibular impairment to delirium remains absent rather than merely limited, and we present this link as a hypothesis warranting dedicated study, not as an established pathway.

It is reasonable to ask whether any evidence directly links vestibular dysfunction to delirium, and the absence noted above should be read literally rather than as a rhetorical qualifier: to our knowledge, no published cohort or trial has tested vestibular status as a predictor of incident delirium. Indirect support instead comes from two adjacent bodies of evidence. First, other sensory impairments that plausibly share mechanisms with vestibular loss, such as reduced environmental monitoring, communication difficulty and disorientation, are established delirium risk factors: multisite hospital data show that dual sensory (hearing and visual) impairment, though not hearing or visual impairment alone, is independently associated with delirium (Morandi et al., 2021). Second, a growing clinical literature associates vestibular dysfunction with cognitive domains attention, executive function and visuospatial processing that are also vulnerable during delirium; longer duration of vestibular-related dizziness has been associated with lower performance on standard cognitive screening in older patients (Ma et al., 2025). Neither line of evidence demonstrates that vestibular impairment itself precipitates delirium, and we present the vestibular-delirium link strictly as an extrapolation from these two adjacent evidence bases pending the direct, prospective study called for in Stage 2 of the research agenda below.

## Dual-task gait as a stress test

Dual-task gait testing evaluates walking whilst the person simultaneously performs a second task. Two broad paradigm families are relevant here, and they probe different mechanisms. Attention-demanding cognitive dual tasks such as serial subtraction, category or letter fluency, or auditory reaction tasks load executive and attentional systems whilst the person walks, and are the paradigm most directly relevant to the cognitive-load component of the proposed model because they approximate the divided attention required during real-world conversation, navigation or hazard monitoring. Manual or motor dual tasks such as carrying a tray or object load concurrent motor planning rather than executive attention, and are informative for coordination but less directly relevant to the cognitive-vestibular interaction proposed here. Studies applying the proposed model should specify which paradigm family is used, since the two are not interchangeable (Table 1).

**Table 1:** Compact conceptual framework for silent neurovestibular frailty in older adults, with evidentiary status indicated for each domain.

Domain	Indicators and evidence	Evidentiary status	Clinical implication
Vestibular vulnerability	Dizziness, imbalance, oscillopsia, visual dependence, falls during turning and difficulty walking in darkness.	Established—balance and vestibular dysfunction are prevalent and functionally important in older adults (Agrawal et al., 2009; Lin and Bhattacharyya, 2012).	Actively screen vestibular symptoms during fall-risk assessment and refer for targeted vestibular evaluation when symptoms or context suggest sensorimotor instability.
Cognitive-vestibular interaction	Impaired spatial orientation, attentional overload, visuospatial difficulty and poor navigation.	Plausible inference – vestibular disorders are associated with cognitive deficits and impaired spatial navigation cross-sectionally, including lower MoCA scores with reduced objective vestibular function; predictive value for mobility outcomes is untested (Bosmans et al., 2021; Ma et al., 2025; Smith et al., 2024; Xie et al., 2017).	Treat vestibular dysfunction as a sensory-cognitive contributor to mobility decline, not only as a peripheral balance complaint; supplement general cognitive screening with navigation-specific testing.
Dual-task mobility vulnerability	Slower walking, gait variability, unstable turning, trunk sway and increased dual-task cost.	Established for cognitive-impairment subgroups; dual-task cost must be interpreted alongside task-prioritisation strategy, not as a pure impairment marker (Ansai et al., 2017; Goncalves et al., 2018; Yogev-Seligmann et al., 2008).	Use dual-task gait, with recorded single-task cognitive performance, as a clinic-based stress test of cognitive-motor reserve to guide rehabilitation referral.
Delirium and functional decline cascade	Cognitive impairment, sensory impairment, immobility, medication exposure, acute illness and sleep disruption increase delirium and fall vulnerability.	Established for shared risk factors between falls and delirium, and for dual sensory (hearing and visual) impairment as a delirium risk factor; a direct vestibular-delirium link is speculative and untested (Hshieh et al., 2015; Inouye et al., 2014; Morandi et al., 2021; Oh et al., 2017).	Combine fall prevention, delirium prevention, medication review, mobilisation and sensory optimisation for high-risk patients.
Digital and prevention pathway	Inertial sensors quantify gait variability, turning and activity; daily-life gait quality predicts falls.	Established for gait monitoring predicting falls in general older-adult samples; application to the combined triad proposed here is speculative (Howcroft et al., 2013; van Schooten et al., 2016).	Connect digital biomarkers to actionable care pathways, including vestibular rehabilitation, cognitive-motor training and geriatric optimisation.
Other established fall-risk contributors	Muscle weakness/sarcopenia, social isolation and loneliness, and hearing loss.	Established, largely independently of the proposed triad (Lin and Ferrucci, 2012; Petersen et al., 2020; Rodrigues et al., 2022).	Retain within existing multifactorial fall-prevention frameworks; the proposed triad is additive to, not a replacement for, these factors.

Evidentiary status reflects the strength of support for each row’s specific evidence base, not for the combined silent neurovestibular frailty triad, which remains an untested hypothesis requiring the staged research agenda. This table presents silent neurovestibular frailty as a candidate, hypothesis-generating prevention framework that links vestibular vulnerability, cognitive-vestibular interaction, dual-task mobility, delirium vulnerability and digital monitoring, whilst distinguishing established evidence from plausible inference and speculative extension for each domain, and situating the framework alongside other established, independently supported contributors to fall risk.

**Figure 1:**
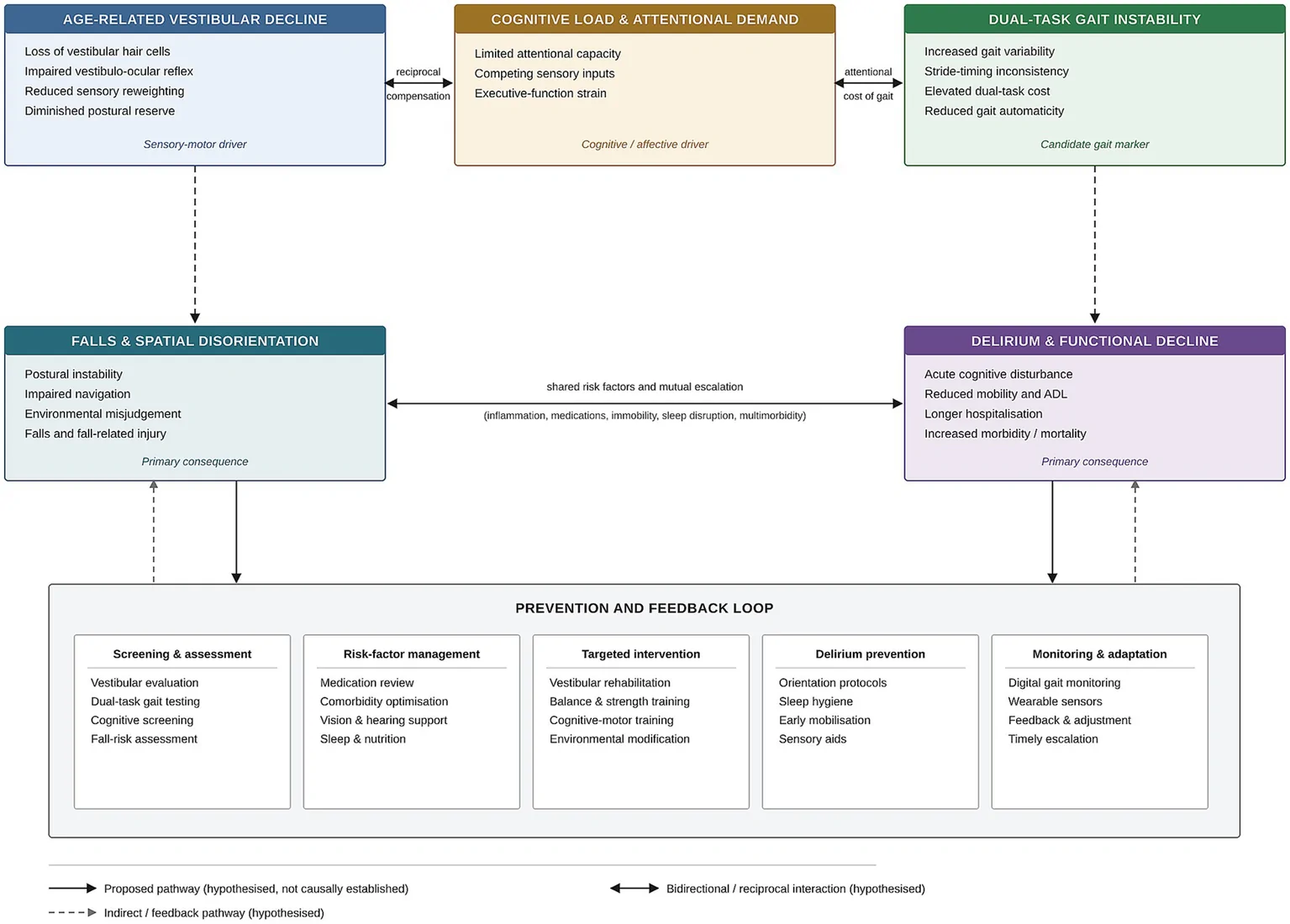
Proposed, hypothesis-generating pathway linking candidate components of silent neurovestibular frailty to falls, delirium and functional decline in older adults. Arrows are drawn as bidirectional where reciprocal influence is proposed rather than a strictly one-directional cascade. Age-related vestibular decline and cognitive load/sensory conflict are shown as mutually reinforcing, and dual-task gait instability is linked bidirectionally to both falls/spatial disorientation and delirium/functional decline. Falls and delirium are shown as mutually escalating outcomes that share risk factors, rather than as a simple sequence. Dashed feedback arrows indicate that prevention and rehabilitation are hypothesised to reduce upstream vulnerability, closing the loop rather than acting only after overt decline has occurred. All pathways depicted are proposed and hypothesised rather than causally established, and none should be read as an established causal chain.

The dual-task cost quantifies the degree to which gait deteriorates when attention is divided, but its interpretation is not straightforward. A large dual-task cost may reflect genuine cognitive-motor impairment, but it may equally reflect a deliberate task-prioritisation strategy in which attentional resources are allocated towards the cognitive task at the expense of gait control, sometimes termed a ‘posture-second’ strategy, rather than an inability to maintain gait (Yogev-Seligmann et al., 2008). Conversely, a small dual-task cost achieved by prioritising gait over the cognitive task may mask cognitive vulnerability rather than indicate its absence. For this reason, studies applying the proposed model should record concurrent single-task cognitive performance and, where feasible, use explicit prioritisation instructions, rather than relying on dual-task cost in gait alone (Figure 1).

Beyond paradigm selection, several methodological design features materially affect dual-task findings and should be specified in any study applying the proposed model. Task difficulty should be calibrated to avoid floor or ceiling effects in either the gait or cognitive outcome, since a task that is too easy fails to reveal interference and one that is too demanding may cause both outcomes to fail uninformatively. Both gait outcomes (e.g., speed, variability, turning) and cognitive-task outcomes (e.g., accuracy, reaction time) should be reported under single- and dual-task conditions, since reporting gait change alone conflates genuine interference with prioritisation. Explicit prioritisation instructions (e.g., ‘walk as you normally would’ versus ‘try to do equally well on both tasks’) should be standardised and reported, since instruction wording alters dual-task cost independently of underlying impairment. Familiarisation trials should precede data collection to reduce novelty-related performance variability, and testing order should be counterbalanced or randomised across participants to avoid systematic fatigue or practise confounds. Fatigue across repeated trials, practise effects across sessions, and the retention and transfer of any dual-task training to untrained task combinations or real-world mobility should be assessed explicitly in intervention studies rather than assumed from single-session performance.

Ansai et al. (2017) showed that gait and dual-task performance differ across older adults with preserved cognition, mild cognitive impairment and mild Alzheimer’s disease. Goncalves et al. (2018) further reported that dual-task performance predicted falls in older people with mild cognitive impairment and mild Alzheimer’s disease. These findings complement evidence that cognitive impairment increases fall risk (Muir et al., 2012), and that the interaction between gait, falls and cognition is sufficiently important to consider cognitive strategies in fall reduction (Segev-Jacubovski et al., 2011).

Dual-task gait is relevant to neurovestibular frailty because vestibular dysfunction is hypothesised to increase the attentional resources required for balance, leaving fewer resources available for conversation, route planning or hazard detection; this mechanism has not yet been directly tested in combined vestibular-dual-task-gait paradigms. Useful clinical and digital variables include gait speed, stride-time variability, step-width variability, turning duration, trunk sway, dual-task cost (interpreted alongside single-task cognitive performance) and task prioritisation. These measures can guide vestibular rehabilitation, cognitive-motor training, medication review, assistive-device reassessment and perioperative delirium prevention, once validated as predictors within this framework.

## Cognitive assessment within the proposed battery

A key remaining question is which cognitive tests should accompany vestibular and dual-task gait assessment, since this has not previously been specified. We recommend a two-tier approach rather than a single instrument. As a first-tier, brief bedside screen, the Montreal Cognitive Assessment (MoCA) is a reasonable default because it is widely validated, freely available, and samples attention, executive function and visuospatial ability alongside memory and orientation (Nasreddine et al., 2005). However, the MoCA was not designed to isolate the visuospatial and navigational sub-domains that appear to be preferentially affected in vestibular dysfunction; in one recent cohort, lower MoCA scores tracked with objectively reduced vestibular function on video head-impulse and caloric testing, with the association most consistent for visuospatial and executive items rather than the total score (Ma et al., 2025). We therefore recommend a second tier of supplementary, domain-specific testing where feasible, focused on spatial navigation and orientation rather than general cognition alone, for example the Triangle Completion Task, on which older adults with vestibular loss perform worse than age-matched controls (Xie et al., 2017), or comparable virtual-navigation paradigms. Reviews of cognition in vestibular disorders support this domain-specific emphasis, reporting the most consistent deficits in spatial memory, visuospatial processing and navigation rather than uniform impairment across all cognitive domains (Bosmans et al., 2021; Smith et al., 2024). Studies applying the proposed model should therefore report both a general screen (e.g., MoCA) and at least one navigation-specific measure, since relying on general screening alone risks under-detecting the specific cognitive signature hypothesised to accompany silent neurovestibular frailty.

## Neuroscience grounding and candidate biomarkers

For an ageing-neuroscience audience, the proposed model should be anchored to measurable mechanisms and candidate biomarkers rather than remaining at the level of clinical description alone. Behavioural gait measures obtainable from instrumented walkways or wearable inertial sensors stride-time variability, step-width variability, turning velocity and duration, trunk sway, and dual-task cost provide accessible, scalable indicators consistent with existing digital gait-monitoring literature (Howcroft et al., 2013; van Schooten et al., 2016).

Neurophysiological indicators offer a complementary, mechanistic layer. Functional near-infrared spectroscopy (fNIRS) studies have shown increased prefrontal cortex oxygenation during dual-task walking relative to single-task walking in older adults, consistent with greater recruitment of executive control resources during cognitively demanding gait (Holtzer et al., 2011). Mobile electroencephalography and vestibular-evoked myogenic potentials represent further candidate measures for probing, respectively, cortical engagement during locomotion and peripheral otolith function.

We add an explicit caveat regarding fNIRS, since its interpretation is frequently overstated. fNIRS measures the haemodynamic correlates of neural activity relative changes in oxygenated and deoxygenated haemoglobin rather than cognitive load or neural activity directly. Its signal is influenced by task design (the specific cognitive task, walking speed and instruction set), extracerebral and systemic physiological signal contamination, motion artefact during walking, and preprocessing and analysis choices, all of which can materially alter reported activation patterns. Any application of fNIRS within this framework should therefore report task parameters, artefact-correction methods and channel placement explicitly, and should treat prefrontal activation as a correlate of, rather than a direct readout of, the cognitive-load construct described above.

At this stage, no single biomarker, behavioural or neurophysiological, can be regarded as validated for the proposed triad. We list these measures as a candidate toolkit for the operationalisation phase of the research agenda outlined later, not as an established assessment battery.

## Vestibular dysfunction as a frailty domain

Vestibular dysfunction is common in older adults but is less routinely screened than vision, hearing, cognition or medication burden. In a nationally representative United States sample, Agrawal et al. (2009) reported a substantial burden of balance and vestibular dysfunction amongst adults, whilst Lin and Bhattacharyya (2012) showed that balance disorders in older adults have meaningful functional impact. Despite this burden, patients may not volunteer dizziness unless specifically asked, and clinicians may attribute unsteadiness to arthritis, neuropathy, general frailty or normal ageing.

We propose that a neurovestibular frailty framework treat vestibular function as a potentially modifiable or compensable domain within fall-risk assessment, rather than as a peripheral complaint, whilst recognising that the degree to which vestibular impairment is modifiable varies by aetiology and has not been established specifically for the population or triad discussed here. Clinical enquiry should include dizziness, oscillopsia, imbalance, motion sensitivity, falls during turning, difficulty walking in darkness, visual vertigo, avoidance of busy environments and medication exposures that worsen dizziness. Bedside assessment may include orthostatic blood pressure, head impulse testing, dynamic visual acuity, positional testing when appropriate, balance testing and gait observation during head turns. Specialist evaluation may include video head impulse testing, vestibular evoked myogenic potentials, caloric testing, posturography or audiovestibular referral.

The purpose of screening, as we envisage it, is not to overdiagnose vestibular disease, but to identify clinically meaningful sensorimotor vulnerability that existing pathways may overlook. Even when vestibular loss is not fully reversible, rehabilitation can improve compensation, gaze stability, balance confidence and functional mobility, based on evidence from the general vestibular rehabilitation literature rather than trials specific to the proposed phenotype. Combining vestibular rehabilitation with strength, balance, cognitive-motor and environmental interventions may be particularly valuable for older adults with recurrent falls, unexplained mobility decline or visually induced dizziness, a hypothesis that intervention trials described later should test directly.

## Delirium, falls and functional decline

Falls and delirium are often managed by different clinical teams, yet they share major risk factors, including advanced age, frailty, cognitive impairment, sensory impairment, polypharmacy, dehydration, infection, pain, sleep disruption and reduced mobility. A fall can trigger hospital admission, fracture management, surgery, opioid exposure, immobility and sleep disruption, each of which may increase delirium risk; delirium may in turn increase fall risk through inattention, agitation, hallucinations, impaired judgement and disturbed gait.

Silent neurovestibular frailty highlights this bidirectional cascade as a candidate explanatory mechanism: vestibular dysfunction may increase the effort required for orientation and movement, cognitive load may destabilise gait, and acute illness may reduce compensatory capacity. We hypothesise that, together, these processes can transform subtle unsteadiness into a fall, delirium and functional decline, though this sequence has not been traced empirically for the specific triad proposed here and remains a target for the prospective studies outlined below.

The prevention implications are nonetheless actionable in the interim. Older adults presenting with falls should be assessed not only for fracture, syncope, medication effects and cardiovascular causes, but also for cognition, delirium risk, vestibular symptoms and dual-task mobility. Older adults admitted with delirium should be treated as high fall risk and should receive mobility assessment before discharge. Evidence that multicomponent non-pharmacological interventions reduce delirium supports integrating delirium prevention with mobility and sensory assessment, independent of whether the specific triad proposed here is subsequently validated (Hshieh et al., 2015).

## Other contributors to fall risk: physical fitness, social isolation and hearing loss

Silent neurovestibular frailty foregrounds three domains, but falls in older adults are multiply determined, and physical fitness, social isolation and hearing loss also warrant explicit comment alongside vestibular, dual-task and cognitive vulnerability, rather than remaining implicit. Reduced muscle strength and sarcopenia are well-established, largely independent contributors to fall risk, and resistance and multicomponent exercise programmes that target strength and postural control reduce fall risk in community-dwelling older adults (Rodrigues et al., 2022), a mechanism already reflected in existing multifactorial guidelines (Gillespie et al., 2012; Montero-Odasso et al., 2022; Sherrington et al., 2019). Social isolation and loneliness are also consistently associated with falls in systematic-review evidence, plausibly through reduced physical encouragement, diminished social support during recovery, and lower engagement in protective activity (Petersen et al., 2020). Hearing loss, distinct from the vestibular dysfunction emphasised throughout this Perspective, is itself an independent predictor of falls; the association persists after adjustment for vestibular balance function, suggesting that hearing and vestibular pathways contribute separately, rather than redundantly, to fall risk (Lin and Ferrucci, 2012).

We do not regard physical fitness, social isolation or hearing loss as less important than the proposed triad. Rather, they are already comparatively well incorporated into existing multifactorial fall-prevention frameworks (Montero-Odasso et al., 2022), whereas vestibular dysfunction, dual-task gait interference and cognitive-load vulnerability are, in our view, comparatively under-assessed as a joint unit in routine practise. Silent neurovestibular frailty is therefore intended as a targeted addition to, not a substitute for, comprehensive multifactorial assessment that already accounts for strength, physical activity, social circumstances and sensory function more broadly; any future operationalisation of the model (Stage 1 below) should specify how it is combined with, rather than in place of, these established factors.

## Clinical and digital integration

A neurovestibular frailty framework is intended to complement, rather than replace, established multifactorial fall prevention. Exercise, balance training, medication optimisation, vision correction, home safety modification and management of orthostatic hypotension remain essential (Gillespie et al., 2012; Montero-Odasso et al., 2022; Sherrington et al., 2019). The added value we propose is identification of a subgroup in whom vestibular dysfunction and cognitive-motor interference are central drivers of risk and who may benefit from combined vestibular rehabilitation and dual-task mobility training rather than generic balance advice alone, a hypothesis requiring the subgroup-comparison studies described in the research agenda below.

In primary care, screening could be embedded into fall-risk assessment through targeted questions about unsteadiness during turning, walking in darkness, slowing or stopping whilst talking, avoidance of outdoor activity because of dizziness, and previous confusion during illness or medication change. In hospitals, high-risk patients may benefit from orientation aids, optimisation of hearing and vision, supervised mobilisation, safe toileting, medication review, hydration, sleep protection, minimisation of unnecessary lines and avoidance of unnecessary sedatives.

Digital phenotyping can extend detection beyond episodic clinical encounters. Inertial sensors can quantify gait speed, variability, turning instability, trunk sway, step regularity, sit-to-stand transitions and daily activity patterns. Howcroft et al. (2013) reviewed inertial sensors for fall-risk assessment in geriatric populations, and van Schooten et al. (2016) showed that daily-life gait quality predicted falls in a one-year prospective cohort. Digital biomarkers proposed for this framework should nevertheless be linked to actionable care pathways and should not widen inequities in frail, rural, low-income or digitally excluded populations.

## Research priorities: a staged empirical agenda

The proposed construct requires empirical testing before it can inform practise, and we structure this as three sequential stages.

### Statistical operationalization

Statistical operationalisation of the proposed triad should not assume a single functional form linking the three domains to outcomes. Candidate specifications include an additive model in which vestibular, dual-task and cognitive-load scores contribute independent, summed risk; an interaction model in which the domains multiply or moderate one another’s effect on falls, delirium or functional decline; a latent-variable (structural equation) model in which a single latent ‘neurovestibular frailty’ factor is estimated from indicators across the three domains and evaluated for measurement invariance across settings; and a latent-profile or latent-class model in which older adults are empirically grouped into qualitatively distinct risk profiles (e.g., vestibular-predominant, dual-task-predominant, combined-high-risk) without assuming a single continuous severity dimension. These specifications are not mutually exclusive and should be compared directly within the same cohort using fit indices, cross-validated predictive performance and clinical interpretability, rather than assumed *a priori*; the additive and interaction models are most readily interpretable for clinicians, whilst latent-variable and latent-profile approaches may better accommodate heterogeneity in how the triad manifests across individuals.

### Stage 1: Operationalisation

Consensus or Delphi work is needed to define measurable indicators and provisional thresholds for each of the three domains vestibular, dual-task mobility and cognitive-load vulnerability analogous to the process used to define cognitive frailty and motoric cognitive risk syndrome (Kelaiditi et al., 2013; Xiang et al., 2022). This stage should specify which dual-task paradigm, which vestibular bedside or instrumented tests, and which cognitive measures (including the general and navigation-specific tiers discussed above) constitute the minimum assessment battery, and should establish inter-rater and test–retest reliability. This stage should also specify how the triad is assessed alongside already-established fall-risk factors such as strength, physical activity, social circumstances and hearing, rather than in isolation from them, and should adopt the dual-task design safeguards (task difficulty calibration, standardised prioritisation instructions, familiarisation, counterbalanced order) described above.

### Stage 2: Prospective validation

Observational cohort studies should estimate the prevalence of combined vestibular impairment, dual-task gait deficit and cognitive vulnerability across community, outpatient, inpatient and long-term care populations, and should determine prospectively whether this triad predicts falls, delirium, hospitalisation, dementia, disability, institutionalisation and mortality better than existing frailty or fall-risk tools used alone. Such studies should include objective vestibular measures, standardised dual-task gait protocols with recorded single-task cognitive performance, validated cognitive assessments and rigorous delirium ascertainment.

Analyses should report incremental validity that is, whether the triad improves prediction beyond established multifactorial fall-risk and frailty instruments rather than merely correlating with outcomes already captured by those tools and should undergo both internal validation (e.g., bootstrap resampling or cross-validation within the derivation cohort) and external validation in independent cohorts and settings before any claim of generalisable predictive value. Because falls and delirium episodes recur and mortality competes with these outcomes, repeated-event or survival modelling (e.g., recurrent-event Cox models, competing-risks analyses) is preferable to single binary-outcome analysis, and missing data arising from attrition, incomplete testing or cognitive impairment itself should be handled with pre-specified methods (e.g., multiple imputation, sensitivity analyses for informative missingness) rather than complete-case analysis alone. Model performance should be reported using both discrimination and calibration statistics, and, where the model is intended to inform practise, decision-analytic measures of clinical utility (e.g., net benefit, decision-curve analysis) against comparator tools, rather than descriptive prevalence or discrimination alone. This stage is also where the vestibular-delirium hypothesis discussed above should be tested directly, rather than inferred from adjacent sensory-impairment or vestibular-cognition literatures.

### Stage 3: Mechanistic and intervention studies

Mechanistic studies should clarify how vestibular loss alters sensory reweighting, attention, spatial navigation and gait adaptation under cognitive load, using the behavioural and neurophysiological candidate measures described above. Intervention trials should test whether combined vestibular rehabilitation, cognitive-motor training and geriatric optimisation reduce falls, fear of falling, gait variability, dual-task cost, delirium incidence and post-acute functional decline, ideally in populations selected using the Stage 1 operational criteria.

Implementation studies should evaluate brief screening pathways for primary care, emergency departments, perioperative clinics and inpatient wards only after Stages 1 and 2 provide evidence of predictive validity, with particular attention to low- and middle-income settings where multimorbidity, underdiagnosed sensory impairment and limited rehabilitation access may converge.

## Discussion

Silent neurovestibular frailty is proposed as a conceptual model and organising heuristic rather than a validated diagnostic phenotype. Its value, if the model withstands empirical testing, would lie in identifying a hidden sensory-cognitive-motor vulnerability that may precede falls, delirium and functional decline. The model is biologically plausible because vestibular signals contribute to balance, gaze stabilisation, orientation, spatial cognition and postural adaptation, and clinically plausible because older adults often fall during multitasking, turning, visual conflict, environmental complexity or acute illness. It is translationally attractive because vestibular rehabilitation, dual-task mobility training, exercise, medication optimisation, delirium prevention and environmental modification are already available interventions that could, in principle, be recombined around this framework.

The central limitation is that direct evidence linking vestibular dysfunction, dual-task gait and delirium into one validated phenotype does not yet exist. The supporting evidence instead comes from adjacent but convergent fields vestibular cognition, fall-risk assessment, cognitive-motor interference, motoric cognitive risk, delirium prevention, sensory-impairment epidemiology and digital gait monitoring (Bosmans et al., 2021; Hshieh et al., 2015; Li et al., 2018; Ma et al., 2025; Morandi et al., 2021; Segev-Jacubovski et al., 2011; Smith et al., 2024; Verghese et al., 2007) each of which supports one or two components of the triad but none of which has tested all three together. We treat this gap as the primary justification for the staged research agenda above, not as a reason to defer clinical attention to the individual, already-supported components.

Care is needed to avoid overmedicalising normal ageing or restricting mobility through fear whilst this evidence is generated. The aim of the proposed model is to preserve safe mobility and cognitive resilience, not to discourage activity or to label older adults as frail prematurely, without waiting for the model to be validated. Older adults identified as vulnerable under existing, validated components of assessment should receive enabling interventions that improve strength, compensation, orientation, confidence and participation. The framework should also remain attentive to social and environmental determinants, including unsafe housing, poverty, limited transport, social isolation, poor access to rehabilitation and caregiver support, which will shape how any future validated version of this model could be implemented equitably.

## Conclusion

Silent neurovestibular frailty offers a conceptual framework for why some older adults appear stable under simple testing conditions yet experience falls, delirium or functional decline when exposed to cognitive load, sensory conflict, environmental complexity or acute illness. We have proposed integrating vestibular dysfunction, dual-task mobility and cognitive reserve into a single candidate risk framework, distinguished this framework from related constructs such as cognitive frailty and motoric cognitive risk syndrome, specified candidate behavioural and neurophysiological biomarkers and a recommended cognitive assessment battery, situated the model alongside other established contributors to fall risk, and outlined a staged empirical agenda statistical operationalisation, prospective validation and intervention testing required before the model can responsibly inform risk stratification.

If validated, this approach would extend established fall and delirium prevention by recognising the vestibular system as a contributor to mobility resilience, spatial cognition and acute brain vulnerability; until then, it should be treated as a research priority rather than a ready-to-use clinical tool. Systematic study across the proposed brain-gait-vestibular axis could ultimately help prevent falls, reduce delirium risk and preserve independence in older adults.

## Data Availability

The original contributions presented in the study are included in the article/supplementary material, further inquiries can be directed to the corresponding author.
